# Incidence of medication errors in a surgical intensive care unit. analisys and improvement measures implemented

**DOI:** 10.1186/2197-425X-3-S1-A75

**Published:** 2015-10-01

**Authors:** P Cuesta-Montero, E Domingo-Chiva, S Plata-Paniagua, JA Monsalve- Naharro, B Montalban-Moreno, JM Jimenez-Vizuete, A Valladolid-Walsh, R Peyro-García

**Affiliations:** Anesthesiology and Critical Care, Complejo Hospitalario Universitario de Albacete, Albacete, Spain; Pharmacy, Complejo Hospitalario Universitario de Albacete, Albacete, Spain

## Introduction

Medication errors in critical care are frequent, serious and predictable. Critically ill patients are prescribed twice as many medications as patients outside the intensive care unit and nearly all will suffer a potential error at some point during their stay.

## Objectives

To quantify and characterize medication errors in a surgical intensive care unit (SICU).

## Methods

We conducted a one-month prospective observational study to detect, quantify and score medication errors in a SICU.

## Results

A total of 634 observations made over weekdays and weekends were performed including morning, noon and night shifts. 36.27 % observations (230) included some type of error, a total of 245 medication errors were detected. According to the type of error found: 52 were prescription errors (21.22%), 2 omissions (0.82%), 44 related to administration technique (wrong speed) (17.96%), 10 omissions of the administration record (4.08%), 97 erroneous preparations (39.59%), 1 wrongly prescribed dose by default (0.41 %) and 3 by excess (1.22%), 5 errors related to erroneous administration route (2.04%), 2 erroneous drug monitorization (0.82 %) and 29 transcription errors (11.84%). According to severity within categories established by the National Coordinating Council for Medication Error Reporting and Prevention (NCCMERP), 26.12% errors were Category A, 10.20% were Category B, 61.63% were Category C, 1.63% Category D and 0.41 % Category F.

## Conclusions

Determining the incidence of medication errors in our system and adopting measures to prevent them is a priority in order to improve the drug treatment process in critically ill patients. The integration of a pharmacist in the intensive care unit allowed us to implement improvement measures to reduce medication related errors and improve quality of care. Measures implemented included: - Development of pharmacotherapeutic protocols and guidelines drug management. - Conducting training sessions for medical staff and nursing.
